# Obesity and Pulmonary Function in African Americans

**DOI:** 10.1371/journal.pone.0140610

**Published:** 2015-10-21

**Authors:** Alem Mehari, Samina Afreen, Julius Ngwa, Rosanna Setse, Alicia N. Thomas, Vishal Poddar, Wayne Davis, Octavius D. Polk, Sheik Hassan, Alvin V. Thomas

**Affiliations:** 1 Howard University, Division of Pulmonary Diseases, Washington, District of Columbia, United States of America; 2 Howard University, Department of Internal Medicine, Washington, District of Columbia, United States of America; Dasman Diabetes Institute, KUWAIT

## Abstract

**Background:**

Obesity prevalence in United States (US) adults exceeds 30% with highest prevalence being among blacks. Obesity is known to have significant effects on respiratory function and obese patients commonly report respiratory complaints requiring pulmonary function tests (PFTs). However, there is no large study showing the relationship between body mass index (BMI) and PFTs in healthy African Americans (AA).

**Objective:**

To determine the effect of BMI on PFTs in AA patients who did not have evidence of underlying diseases of the respiratory system.

**Methods:**

We reviewed PFTs of 339 individuals sent for lung function testing who had normal spirometry and lung diffusion capacity for carbon monoxide (DLCO) with wide range of BMI.

**Results:**

Functional residual capacity (FRC) and expiratory reserve volume (ERV) decreased exponentially with increasing BMI, such that morbid obesity resulted in patients breathing near their residual volume (RV). However, the effects on the extremes of lung volumes, at total lung capacity (TLC) and residual volume (RV) were modest. There was a significant linear inverse relationship between BMI and DLCO, but the group means values remained within the normal ranges even for morbidly obese patients.

**Conclusions:**

We showed that BMI has significant effects on lung function in AA adults and the greatest effects were on FRC and ERV, which occurred at BMI values < 30 kg/m2. These physiological effects of weight gain should be considered when interpreting PFTs and their effects on respiratory symptoms even in the absence of disease and may also exaggerate existing lung diseases.

## Introduction

Obesity is associated with increased morbidity, and mortality. The prevalence of obesity [as indicated by a body mass index (BMI) of ≥30 kg m^2^] has increased in the US during the last several decades. It has increased from 15% in 1976–80, to 23% in 1988–94, to 32% in 2003–04[1,2]. More than one-third (age-adjusted 34.9%) of US adults are now obese [3,4] and is projected to rise to over 50% by the year 2030 [5]. While the overall prevalence of obesity has increased, significant racial/ethnic disparities continue to exist; with highest prevalence among non-Hispanic black (47.8%) than non-Hispanic white (32.6%) adults [3].

The mass loading effect of adiposity on the thoracic cage and abdomen can have an effect on respiratory mechanics leading to decreased chest wall and lung compliance, decreased pulmonary gas exchange [6], limitations in exercise capacity [7–9] and altered pulmonary physiology [9–11].

Although the impact of obesity on PFTs has been addressed in previous studies [10,12–14], many of the previous studies have been small; or they were conducted with the subjects in the supine position [10], some studies [15–17] reported only spirometry results or they included only two BMI groups [13,18] and the study which included a wide range of BMI was done in exclusively white patients [11]. However, a study looking at the association of full lung function with wide range of BMI in AA patients is limited.

In our experience, we often encounter obese black patients reporting respiratory symptoms such as dyspnea and we hypothesized they will have abnormal lung function. Therefore, in this study we aim to determine the effects of obesity on PFTs in a large healthy African American subjects who exhibited a wide range of BMI.

## Methods

The data used were collected from PFTs performed between 2008 and 2013. The most common indication for PFT was dyspnea. Participants performed spirometry, body plethysmography, and lung diffusion capacity for carbon monoxide (DLCO) according to American Thoracic Society/European Respiratory Society (ATS/ESR) standard protocols [19,20]. At the study visit weight was measured using a calibrated scale and height was measured with patients not wearing their shoes, using a calibrated stadiometer. The measurements of weight and height were used to calculate BMI and the BMI was categorized into 5 groups (see below) based on the established standard [21]. This study was conducted in accordance with amended Declaration of Helsinki. The institutional review board of Howard University approval was obtained and the requirement of informed consent was waived. All PFTs performed during the study period were reviewed. The PFTs not selected for this study were either having significant abnormality or patients had known cardiopulmonary disease. Patients comprising this study had the following characteristics: (1) age≥18 years, (2) smoking history of < 10 pack-years, (3) BMI ≥ 20 kg/m^2^ (4) black race (5) no known diagnosis of cardiopulmonary or chest wall disease, (6) expiratory volume in 1 second (FEV1) to vital capacity (FVC) ratio (≥0.7), (7) DLCO ≥80% of predicted after adjusting for the patient’s hemoglobin.

Spirometry was performed, to obtain FVC, FEV1, and FEV1/FVC ratio and values expressed as % predicted [22] except for the FEV1/FVC ratio where the actual measurement was used. Lung volumes were measured using a body plethysmograph that was calibrated daily (Vmax version v21-2B; Yorba Linda, CA, USA) according to ATS/ERS Guidelines for lung volume testing [23]. Values derived were vital capacity (VC), functional residual capacity (FRC), total lung capacity (TLC), residual volume (RV), and expiratory reserve volume (ERV) and were reported as percentage of predicted values [24]. Predicted ERV was obtained by subtracting the predicted RV from the predicted FRC. TLC was determined by FRC plus inspiratory capacity, and RV was determined by TLC minus VC. DLCO was adjusted for hemoglobin concentration and expressed as percent of predicted.

We subdivided the patients characteristics and PFT results into BMI categories of 20 to 24.9 kg/m^2^ (normal weight or category 1), 25 to 29.9 kg/m^2^ (over weight or category 2), 30 to 34.9kg/m^2^ (mild obesity or category 3), 35 to 39.9 kg/m^2^ (moderate obesity or category 4), ≥40kg/m^2^ (morbid obesity or category 5) [21].

Descriptive statistics were computed to assess patient baseline demographics and PFTs for the different BMI categories. Analysis of variance (ANOVA) was performed to assess significant differences in means among the BMI categories with a Benferroni *post Hoc* analysis. The results were analyzed using multiple linear and polynomial regressions to assess the effects of BMI on lung volumes. Significance was taken as p value < 0.05 for all tests. Data analysis was conducted using the Statistical Analysis System software 9.3 (SAS Institute, Cary, NC) and Statistical Analysis and Graphics (NCSS 9.0.7, Kaysville, UT).

## Results

Patient characteristics and mean values for FEV1/ FVC ratio, lung volumes and DLCO for the different BMI categories are summarized in Table 1 and S1 Fig. Age ranged from 18 to 87 years (median 47years) and BMI ranged from 20.05–64.38 kg/m^2^ (median 28.15kg/m^2^). The different BMI categories were well matched for gender except for the two highest BMI categories (moderate and morbid obesity); however, there were no significant differences in the best-fit regression lines between men and women for the effects of BMI (kg/m^2^) on TLC%, VC%, RV%, FRC%, ERV%, or DLCO%. Therefore, the data from men and women have been grouped together. The lung function values were expressed as mean percentages of predicted values with FEV1/FVC, RV/TLC and FRC/TLC expressed as absolute ratios.

**Table 1:** Demographics and Pulmonary Function Results for the Different BMI Groups (Kg/m^2^).

	Normal Weight	Over Weight	Mild Obesity	Moderate Obesity	Morbid Obesity
	BMI = 20–24.9	BMI = 25–29.9	BMI = 30–34.9	BMI = 35–39.9	BMI ≥ 40
**Variables**					
Age (yr.)	44.88 (17.41)	47.43 (14.99)	49.16 (13.33)	49.80 (12.89)	40.54 (10.96)
BMI-kg/m^2^	22.71 (1.29)	27.30 (1.37)	32.27 (1.39)	36.73 (1.14)	47.02 (5.90)
Gender					
Female	50	46	31	24	30
Male	51	54	33	11	9
FEV1/FVC	81.84 (7.41)	81.74 (5.28)	83.47 (4.60)	83.26 (4.62)	85.31¥ (4.11)
VC %	92.53 (12.13)	93.05 (11.89)	93.84 (13.04)	89.17 (9.12)	95.03 (12.29)
TLC%	93.99 (13.19)	95.05 (10.64)	96.53 (14.10)	91.63 (9.40)	93.77 (13.57)
RV%	96.36 (34.11)	98.42 (24.92)	101.63 (30.43)	96.33 (18.70)	90.78 (26.00)
FRC%	86.69 (16.38)	82.31 (15.10)	78.96 (19.46)	72.35 (11.44)	67.10 (15.09)
ERV%	73.64 (32.55)	58.74 (27.89)	44.87 (20.30)	35.28 (15.89)	33.42 (18.13)
DLCO%	92.69 (12.07)	90.02 (9.65)	93.61 (12.31)	87.49 (9.60)	82.85 (3.77)
RV/TLC	0.38 (0.17)	0.42 (0.33)	0.38 (0.13)	0.38 (0.10)	0.34 (0.10)
FRC/TLC	0.60 (0.20)	0.60 (0.41)	0.50 (0.16)	0.47 (0.13)	0.44 (0.12) ¥

* Values are expressed as No. or mean percentage of predicted (SD), RV/TLC and FRC/TLC are expressed as absolute ratios. Comparisons between BMI groups for ERV, FRC, and DLCO are shown in S1 Fig.

¥For FRC/TLC and FEV_1_/FVC, P<0.05 compared to BMI groups 20–24.9 and 25–29.9kg/m^2^

BMI = body mass index

The mean values for VC% predicted, TLC% predicted, RV% predicted remained within the normal range for the different BMI categories. The RV/TLC ratio did not change significantly between any of the BMI categories, but the FRC/TLC ratio and FRC decreased significantly between categories indicating that FRC is affected by BMI. The preservation of TLC in the presence of decreasing FRC with increases of BMI can be explained due to increase in inspiratory capacity. The slow VC was also remained unchanged with increases of BMI, however we noticed the FVC was lower than the slow VC and this could be due to airway compression during the forced maneuver. For FEV1/FVC ratio, although the mean of the BMI groups remained within the normal limits, the morbidly obese category had significantly higher FEV1/FVC ratio when compared to those with normal (p = 0.014) and overweight (p = 0.011) showing the obese group having lower mean FVC% predicted. The mean values of DLCO was within normal limits even in the morbidly obese category but showed a significant decrease with the increases of BMI. The morbidly obese category had a significantly lower mean DLCO when compared to those with normal BMI, over weight and mild obesity BMI categories (p<0.001, p = 0.004, and p<0.001 respectively).

**Fig 1:**
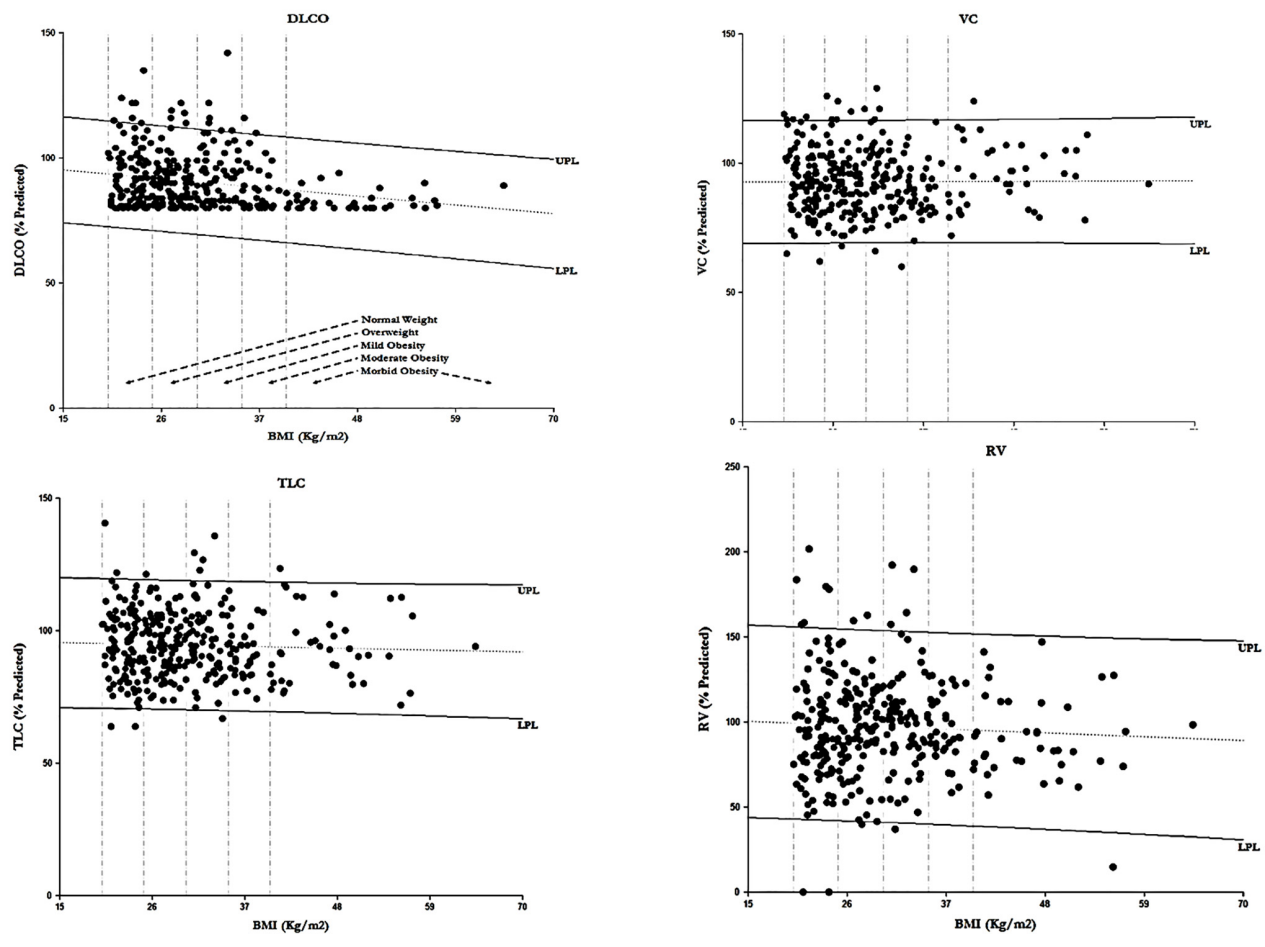
The linear regression between BMI and DlCO (top left), VC (top right), TLC (bottom left), and RV (bottom right). Linear regression between BMI and DLCO (P < .0001), VC (P = 0.895), TLC (P = 0.459) and RV (P = 0.301). The intervals represent the lower prediction limit (LPL) and the upper prediction limit (UPL) of the regression line. The vertical dash lines are the cut-points separating the BMI categories defined in the DLCO graph. The slopes of the linear regression for percentage of predicted values and BMI were -0.166 (DLCO), 0.005 (VC), -0.026 (TLC), and -0.016 (RV).

Fig 1 shows the linear regressions for DLCO, VC, TLC, and RV. In all cases but DLCO, there was no significant linear relationship with BMI. The p-value of the linear regression for DLCO was < .0001. The lower prediction limit (LPL) and the upper prediction limit (UPL) of the regression line were plotted with some patients having values outside the prediction limits. The slopes of the linear regression for percentage of predicted values and BMI were -0.166 (DLCO), 0.005 (VC), -0.026 (TLC), and -0.016 (RV).

**Fig 2:**
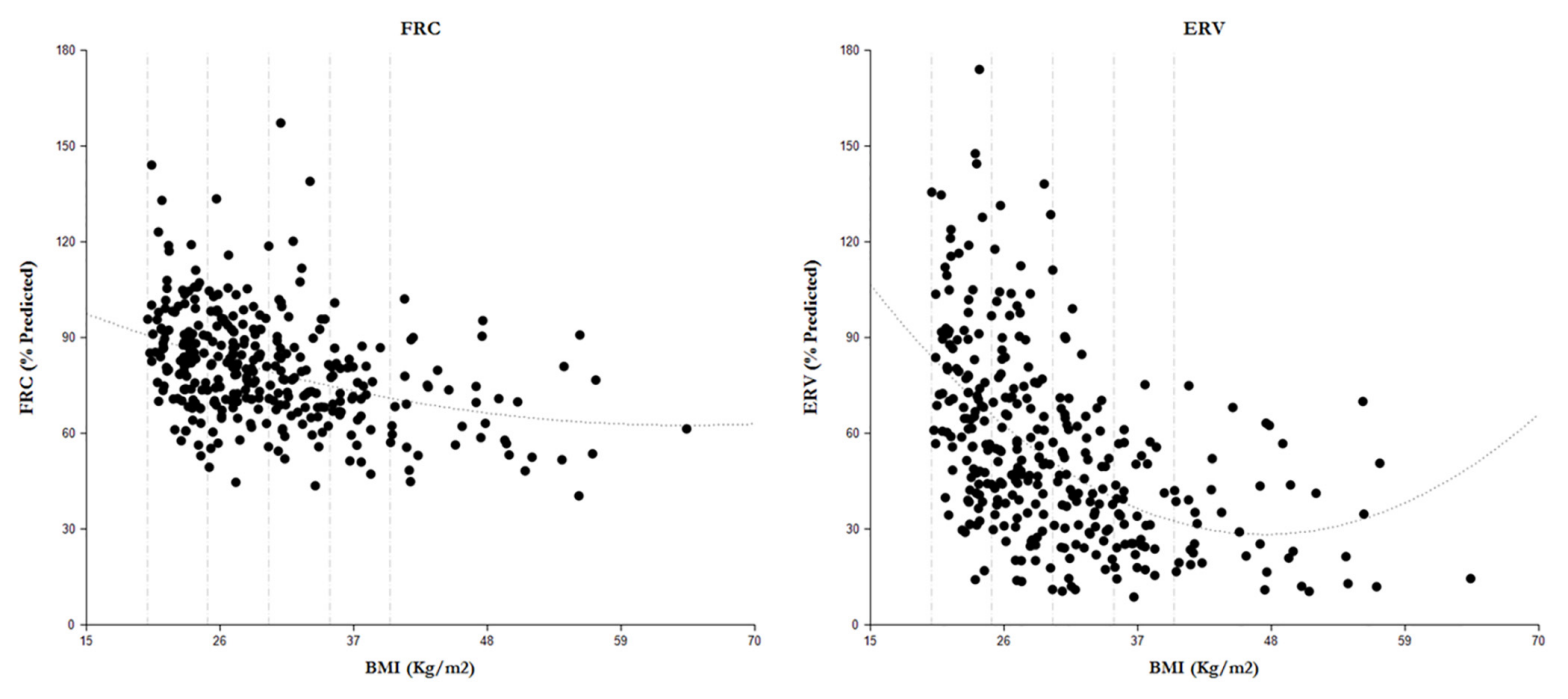
The polynomial regressions for FRC (left) and ERV (right). The r^2^ values for FRC and ERV were 0.151 and 0.269, and the second order polynomial regression equations were as follows: FRC = 0.0143*(BMI)^2^–1.8448*(BMI) + 121.88 and ERV = 0.0742*(BMI)^2^–7.0384*(BMI) + 195.29 with p < 0.0001. The BMI classifications are the same as those in Fig 1.

Multiple linear regression analysis showed a dramatic decrease in ERV as BMI increased. When compared to those with a normal BMI, ERV decreased by 14.08%, 27.4%, 38.61% and 43.7% in the overweight, mild obesity, moderate obesity and morbid obesity respectively (Table 2). An equally consistent negative correlation between BMI and FRC was also observed, although the changes are less dramatic. When compared to those with a normal BMI, FRC reduced by 4.7%, 8.8%, 16.2% and 20% in the overweight, mild obesity, moderate obesity and morbid obesity respectively (Table 2). The best-fit polynomial regressions for FRC and ERV were highly significant (Fig 2) with p<0.0001. The second order polynomial regression equations were: FRC = 0.0143*(BMI)^2^–1.8448*(BMI) + 121.88 and ERV = 0.0742*(BMI)^2^–7.0384*(BMI) + 195.29. The r^2^ values for FRC and ERV were 0.151 and 0.269 respectively. The results indicate that both FRC and ERV decrease sharply with increase in BMI values.

**Table 2:** Multiple Linear Regressions Modeling for the Association of BMI (kg/m^2^) and Lung Function.

	Normal	Over Weight		Mild Obesity		Moderate Obesity		Severe Obesity	
	BMI = 20–24.9	BMI = 25–29.9		BMI = 30–34.9		BMI = 35–39.9		BMI> 40	
Variables		β (SE)	P value*	β (SE)	P value*	β (SE)	P value*	β (SE)	P value*
FEV_1_/FVC	Ref	0.15 (0.77)	0.850	1.99 (0.88)	0.030	1.36 (1.09)	0.210	2.46 (1.05)	0.020
VC%	Ref	1.09 (1.54)	0.480	1.95 (1.76)	0.270	-4.44 (2.18)	0.040	-0.53 (2.09)	0.800
TLC%	Ref	1.20 (1.73)	0.490	2.30 (1.97)	0.240	-3.55 (2.43)	0.150	-1.55 (2.34)	0.510
RV%	Ref	1.35 (3.96)	0.730	3.28 (4.50)	0.470	-1.07 (5.57)	0.850	-3.26 (5.37)	0.550
FRC	Ref	-4.66 (2.23)	0.040	-8.79 (2.54)	<0.001	-16.24 (3.14)	<0.001	-19.76 (3.03)	<0.001
ERV	Ref	-14.09 (3.62)	<0.001	-27.4 (4.12)	<0.001	-38.61(5.09)	<0.001	-43.66 (4.91)	<0.001
DLCO	Ref	-2.68 (1.47)	0.070	1.05 (1.68)	0.530	-4.37 (2.08)	0.040	-9.18 (2.00)	<0.001
RV/TLC	Ref	0.02 (0.03)	0.390	-0.02 (0.03)	0.650	-0.01 (0.04)	0.940	0 .01 (.038)	0.900

**Model**: Lung Function = BMI + Age + Gender

**Abbreviations**: BMI = Body Mass Index; SE = Standard Error.

Coefficients are expressed as change in percent predicted lung function per unit BMI (kg/m^2^)

*P value less than 0.001 were deemed significant, after the Bonferroni adjustment

## Discussion

The result of this study confirms as well as reports new findings on the effects of obesity on lung function in AA adults. Among the variables, FRC and ERV had the most expressive values, decreasing with the increases of BMI as shown in previous studies [10–14]. However, our study shows the effects of BMI on lung volumes in blacks, information which has never being reported from any of the previous studies [10–12,14,18] that included small number and limited BMI groups or the largest study with wide range of BMI included only white patients [11].

There was an exponential relationship between BMI, ERV and FRC with a reduction in FRC and ERV detectable even in overweight individuals. In a regression analyses, for a person with BMI of 20Kg/m2, FRC and ERV were 90.70% and 84.20% respectively. While for a person with BMI 30Kg/m2, FRC and ERV are 79.41% and 50.92% respectively. Therefore, the FRC of a person with a BMI of 30 Kg/m^2^ is only 88%; and ERV is only 60% of the values for a person with a BMI of 20Kg/m2. So, we see a drastic fall in FRC and ERV even when a person is at the borderline between overweight and mild obesity. The exponential decrease in FRC and ERV is similar to the findings of Jones et al. [11], however the absolute effect of BMI on FRC and ERV in their study was greater than in our patients. Similarly the absolute effect of BMI on FRC shown by Pelosi and colleagues [10] was much higher than Jones et al and our patients. However, Pelosi et al performed their studies on supine and anesthetized and Jones et al studied only white and ours are all black patients.

Some of the differences between the Jones and ours can be due to the race ethinicity difference in both studies. It is well known AAs have relatively longer lower extremities than did white subjects that in turn might influence BMI-fatness relations. Studies have shown, the same BMI categories are associated with lower levels of body fat in non-Hispanic blacks than in other race-ethnic groups [25–27] and health risks associated with a given BMI level may be lower for blacks than for whites [28–31], however, if this holds true for lung function merits further study using more accurate measures of body composition.

We found the effects of obesity on the extremes of lung volumes, at total lung capacity (TLC) and RV to be modest and none significant and VC was unaffected with increasing BMI. Many other studies report an association between increasing body weight and decreasing TLC [11,16,32], however, the changes are small, and TLC is usually maintained above the lower limit of normal, even in severe obesity [11,12,16,33,34]. Similarly the RV and the RV-to-TLC ratio remained normal as reported by others [16,35–37]. In the presence of well-preserved RV, the reduction in FRC can be explained by marked decrease in the ERV[11]. ERV can decline further in the supine position as the diaphragm assumes a higher position in the chest [38].

The observed reduction of FRC and ERV with increasing BMI has clinical implications. There is consistent evidence that, because the FRC is so low in obesity, closing capacity exceeds the FRC, and airway closure can occur within the tidal breaths [39–44] leading to maldistribution of ventilation and impaired gas exchange and hypoxemia [39,40]. In obese individuals, distribution of normal regional ventilation may be reversed [45–47]. Holley et al.[46] found that, in obese subjects with marked reductions of ERV to <20% predicted, ventilation was preferentially distributed to the upper zones of the lung, leaving the lower, dependent zones relatively under ventilated. The reduction in FRC and ERV in obesity is also associated tidal expiratory flow limitation (EFL), especially in the supine [44,48]. The presence of tidal EFL and/or airway closure can be a risk of peripheral airway injury [42] and this may be one of the potential mechanisms explaining the observed link between obesity and bronchial asthma [42,43,49] and the complaints of dyspnea that we often encounter in our obese patients in clinical practice.

The DLCO in this study remained with in normal limits even in the morbidly obese but decreased as BMI increased. The effect of obesity on the DLCO in many studies was reported to be normal, [11,14,50,51] even in morbid obesity [35]. However, some studies suggest it may be increased in extremely obese subjects [11,14,18] probably as a result of the increase in blood volume [11,14,35,52]. Even though the mean predicted values of subjects grouped by degree of obesity were within the normal limits the DLCO decreased as BMI increased. However the decrease was minimal and was seen in the extremes of obesity. The reductions in DLCO with extremes of obesity in this study can be due the effects of reduced FRC and ERV leading to a ventilation perfusion (V/Q) mismatch and micro atelectasis that occur as BMI increases but can also be due other undiagnosed cardiorespiratory illnesses such as pulmonary embolism and pulmonary hypertension which commonly occur in obesity and are known to reduce DLCO.

In summary in this study we confirm the most consistent finding s of the effects of obesity on lung volumes which is the reductions in FRC and ERV in black patients. We also report for the first time the absolute effects of increasing BMI on FRC and ERV is much lower in blacks when compared to the reported data in whites [11]. Contrary to the findings of any other study we also report a decreasing in DLCO with increases of BMI. However our study is not without limitations. First BMI is used as a proxy of obesity but BMI measurements lack the ability to differentiate between lean, fat mass and the distribution of fat which might have a deferential effect on lung volumes. Consequently, a higher BMI may not predict the physiological changes on the lung function universally. Studies have shown that dual energy X-ray absorptiometry (DEXA) had significantly better correlation with lung function impairment than anthropometric measurements [50]. In NHANES, however, BMI was found to be highly correlated with percentage body fat as measured by DEXA [27]. USA and international definitions of overweight and obesity for adults are also based on BMI. Standard BMI categories are well established and widely used and most studies that have examined the effects of obesity on lung function have used BMI. Moreover, this was a cross-sectional study and a cause and effect relationship between obesity and decreased lung function could not be inferred. Another limitation is that our entire patient population was black, and the findings may not translate to other ethnic groups.

### Conclusion

With the epidemic of obesity, health care providers will often encounter obese patients to report greater respiratory symptoms requiring PFTs. Physiologically, increasing weight gain is associated with lung volume reduction even in the absence of specific respiratory disease, and may also exaggerate the effects of existing lung disease. Therefore it is important to consider the physiological changes associated with obesity when interpreting PFTs.

## Supporting Information

S1 Dataset (XLSX)

S1 Fig Effects of BMI on FEV1/FVC (top left), DLCO (top right), ERV (bottom left) and FRC (bottom right). The horizontal solid lines are significant differences between-group comparisons from ANOVA and post hoc test.
(TIF)
