# Supplementary figures and images for: Obesity and Pulmonary Function in African Americans
Source: PLoS One. 2015 Oct 21;10(10):e0140610. doi: 10.1371/journal.pone.0140610 (PMC4619259; doi:10.1371/journal.pone.0140610)

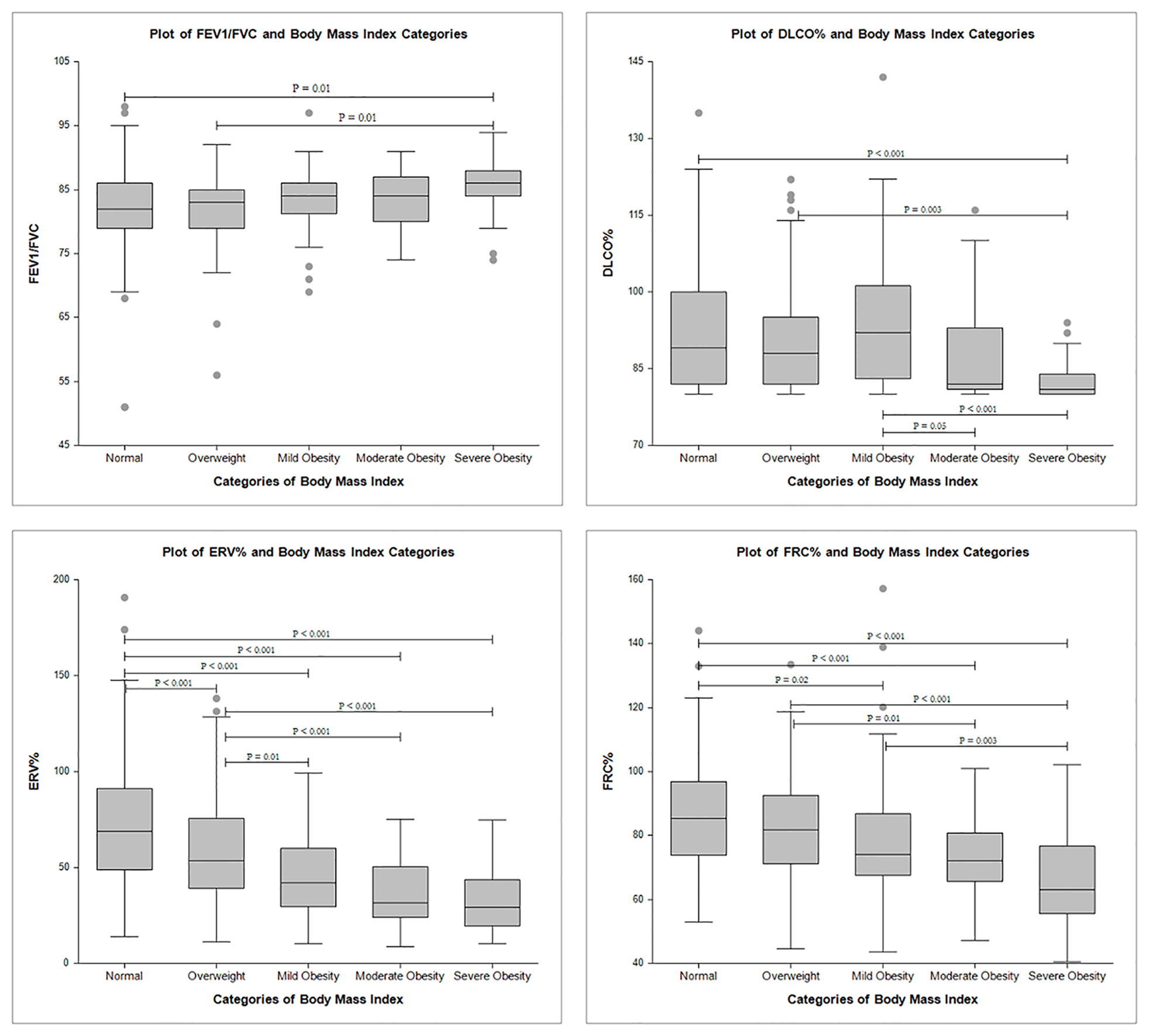

Supplement: S1 Fig — The horizontal solid lines are significant differences between-group comparisons from ANOVA and post hoc test. (TIF) [file pone.0140610.s002.tif]
